# Syphilis and Locomotor Ataxia

**Published:** 1882-07

**Authors:** 


### ORIGINAL TRANSLATIONS AND ABSTRACTS.

### SYPHILIS AND LOCOMOTOR ATAXIA.

In the *Monatsblætter für Praktische Dermatologie*, for June, Dr. E. Remak, of Berlin, gives an exhaustive review of the recent literature upon the relations of locomotor ataxia and syphilis, which we consider of sufficient importance to translate entire.

Since Fournier * first gave prominence to syphilis as one of the most frequent causes of locomotor ataxia, and Vulpian † a few years later also called attention to the great frequency of syphilitic antecedents in tabetic patients, the question of the direct and indirect relations of locomotor ataxia to syphilis has continued to occupy the attention, especially of neurologists. In 1880 a considerable amount of literature upon the subject had already accumulated, which was reviewed by Mœbius.‡ This literature has since then been largely added to. In spite, however, of this evidence of active interest it may be stated in advance that there does not exist unity of opinion among the neurologists upon the question. A minority declare unconditionally for a syphilitic locomotor ataxia, a number assume an ataxic predisposition among syphilitic subjects, while finally a considerable number deny any causative relation between the two diseases altogether.

The advocates of a syphilitic tabes have not shown the existence of any peculiar symptoms or pathological conditions, but base their opinions essentially upon statistical data, and more rarely upon the coexistence of syphilitic disease of other organs, or the therapeutic results of anti-syphilitic treatment.

---

* De l'ataxie locomotrice d'origine syphilitique. Leçons receuillies par Dreyfous, Paris, 1876.

† Leçons sur les maladies de la moelle, 1879.

‡ Schmidt's Jahrbücher der Medicin, Bd. 187. Heft. 3.

The statistics of most authors are limited to the selection from their records of cases of locomotor ataxia with and without syphilitic antecedents. The sources of error in following this method are plain, and have been often urged as objections to the method.

Berger * stated that 20 per cent of his cases of tabes were in syphilitics. Erb † found 52 per cent of his first series of 44 cases with syphilitic antecedents. Mœbius ‡ found the percentage of tabetic patients with syphilis reported up to 1880 to be 23.5 per cent. Against this small percentage Erb § raises the objection that only very carefully examined cases were available in arriving at certain conclusions. Erb states that as his material increases the percentage of cases found syphilitic becomes larger. In 100 cases analyzed in the communication above cited, only 12 per cent were found without precedent syphilis or chancre, and 88 per cent with antecedent syphilis or chancre, 59 per cent of which had had secondary syphilitic manifestations. Shortly afterward Erb, ‖ having added 22 cases, arrived at the result that among 100 cases of locomotor ataxia 60 had secondary syphilis, while 30 had been affected with chancre, and therefore might be added to the cases of syphilitic tabes, making 90 per cent of cases of ataxia with antecedent syphilis. Erb goes farther, and admits the probability that in the remaining ten per cent, hereditary syphilis might be assumed as the cause of the ataxia. Against the objection made to his conclusions that his material was gathered chiefly from the higher classes among whom syphilis is very frequent, Erb had already shown that among his dispensary patients with tabes, 75 per cent suffered from secondary syphilis. Of 500 cases of non-ataxic, principally nervous affections, only 23 per cent had ever been infected, 12 per cent with secondary syphilis and 11 per cent with chancre alone. This disposes of the theory of a mere coincidence of tabes and syphilis in his cases.

Although Erb, next to Vulpian, who according to Fournier ¶ has never seen a tabetic patient without specific antecedents, has been the most prominent advocate among German neurologists of the syphilitic etiology of locomotor ataxia, there is no lack of corroborative statistical

---

* Breslauer Aertztliche Zeitschrift, 1879, Nos. 7 and 8.

† Deutsches Archiv fuer klin. Medicin, 24. Bd., 1879.

‡ Berlin. klin. Wochenschrift, 1880, Nos. 10 and 11.

§ Centralbl. f. d. Med. Wissenschaften, 1881, pp., 195 and 213.

‖ Trans. International Medical Congress, 1881, Vol. II., p. 32, et seq.

¶ De l'ataxie locomotrice syphilitique. Ann de Dermatologie et de Syphiligraphie, 1882, Nos. 1 and 2.

testimony. Gowers * found among 33 cases of tabes 70 per cent who confessed to a primary sore or secondary manifestations, and 53 per cent who suffered from undoubted constitutional syphilis. Althaus,† who found syphilitic antecedents in only 3.9 per cent in patients suffering from neuralgia, 6.2 per cent in hemiplegics and 4.8 per cent in epileptics, found 90.6 per cent of his ataxic patients syphilitic, without, however, admitting a syphilitic ataxia. Voigt ‡ found in 43 cases of locomotor ataxia in men 29, or 67 per cent with syphilis, which he regards as rather below than above the true proportion.

Opposed to these results are the statistics of Benedikt, § according to whose figures only a small percentage of ataxic cases occurs in syphilitics. Rosenthal ‖ also observed among 65 cases of tabes (records of older date) only one after syphilitic infection. In a careful examination of 105 more recent cases he found 18 per cent with syphilitic antecedents. Bernhardt,¶ who formerly found 21.5 per cent of his tabetic cases in syphilitics, found the percentage to rise to 45 in a later series of cases.

These cases, considered without reference to further details, seem to confirm the very frequent occurrence of tabes in men with syphilitic antecedents. It is generally agreed, however, that the co-existence of syphilis and ataxia is much less frequently established in women. The advocates of the syphilitic etiology of ataxia refer this to the much greater difficulty of eliciting a syphilitic history in women.

These and all statistics are, however, held to be incapable of demonstrating the existence of asyphilitic ataxia by very high authorities, among whom may be especially mentioned Westphal,** Lancereaux and Leyden. These authorities hold that a scientific demonstration of the relations of syphilis to ataxia can only be accepted when the pathologico-anatomical evidences of syphilis can be shown to be present, which has not yet been done. At post mortem examinations of individuals dead with locomotor ataxia, syphilitic alterations of other organs have been so rarely found that a syphilitic pathogenesis of the typical degeneration of the posterior columns could not be accepted. The typical degeneration of the posterior columns found in tabes, according to Juillard,†† differs markedly from the circumscribed syphilitic processes which occur in the brain and spinal cord just as in other organs. Erb, however, insists that it is not yet

* Lancet, Jan. 15, 1881.

† Lancet, Sept. 17, 1881.

‡ Berl. klin. Wochenschrift, No. 39, 1881.

§ Wiener Med. Presse, 1881.

‖ Ibid., 1881.

¶ Deutsche Med. Wochenschrift, No. 1, 1882.

** Archiv, f. Psych. und Nervenkr., 1881, p. 230, et seq.

†† Etude critique sur les locatisations spinales de la syphilis, Paris, 1879.

established that syphilis may not affect systems as well as localized parts of a system. Erb also questions the propriety of considering tabes as a disease affecting the spinal system alone, inasmuch as many symptoms in tabes indicate the involvement of certain of the cerebral nerves. Erb has also found syphilitic affections of the skin and mucous membranes more frequently in ataxia, and in eight autopsies of tabetic cases noted syphilitic alterations of other organs in three cases.

According to Benedikt, cases of syphilitic tabes pursue an irregular course (marked variation in the manifestations, unusual beginning, e.g., with sciatica or large anæsthetic patches of the thighs). On the other hand, Erb and Voigt were unable, on a careful comparison of the various symptoms, to detect any difference between the symptomatology of syphilitic and non-syphilitic cases of locomotor ataxia.

The most ardent advocates of the syphilitic causation of tabes do not claim any great success from anti-syphilitic treatment in this disease. Most of them only assert an amelioration of certain symptoms, while decided improvement or cure at all events are among the greatest rarities. Berger, Müller * and Rumpf † have reported such cases, which must be judged with great caution, however, in view of the peculiar spontaneous remissions occasionally noticed in cases of locomotor ataxia. Benedikt, Rosenthal, Leyden and the writer agree in stating that cases of ataxia have become decidedly worse under anti-syphilitic treatment, and that no method is so successful in rapidly reducing the strength of tabetic individuals as an energetic inunction cure. The advocates of the syphilitic causation of tabes, however, refer the want of success of syphilitic treatment to the complete atrophy of the nerve-tissue when the treatment is begun.

This controversy on the part of the neurologists has until now only called forth two contributions from syphilologists—the work of Fournier before cited and one from the pen of Reumont. ‡ The latter analyzed 3400 cases of syphilis, mostly in advanced stages of the disease. Of these 290, or 8.5 per cent had some disease of the nervous system which was in more or less close relation to the syphilis. 14.4 per cent of the 290, or 1.6 per cent of the total number of syphilitics suffered from locomotor ataxia. Reumont believes that this percentage would be greater if at the time when most of these patients were under observa-

* Symptomatologie und Therapie der Tabes dorsalis im Initialstadium, Graz, 1880.

† Berlin. klin. Wochenschrift, No. 36, 1881.

‡ Syphilis und Tabes dorsalis, Aachen, 1881.

tion, the symptomatology of locomotor ataxia, especially in the earlier stages, had been better known. He believes that many beginning cases of ataxia had escaped observation or had been included under the head of some other affection. But certain reservations seem to be necessary before adopting this author's conclusion that syphilis is a prominent factor in the causation of ataxia, since other recognized causative elements of this disease were present in a number of the cases, such as venereal excesses, intemperance, etc.

Among the clinical observations of Reumont the greater frequency of syphilitic symptoms of the skin, the mucous membranes and the bones in his tabetic cases, are of interest. These coincident syphilitic manifestations were present in 14 out of 36 cases.

The results of treatment (antisyphilitic) which Reumont claims to have obtained would be of more interest if the diagnosis of locomotor ataxia could be accepted in all of his cases. Of 36 cases, two are reported cured, 13 improved, and 21 not improved. In 14 of these cases syphilitic symptoms were still present when beginning treatment. It is worthy of remark that this was the case in the two cases reported cured and six of those noted as improved. The author further states that those cases which ran an irregular course (perhaps not strictly locomotor ataxia), and which presented syphilitic manifestations when beginning treatment, gave the best results.

Reumont comes to the practical conclusion that a specific course of treatment in cases of ataxia with syphilitic antecedents is proper, independent of the presence or absence of syphilitic symptoms at the time. On the other hand, in advanced cases in which the strength of the patient has begun to give way, syphilitic treatment, especially a mercurial course, is likely to do harm.

The recent article of Fournier * contains little that is new, but groups neatly all the grounds in favor of the connection of syphilis and locomotor ataxia. The author first disposes of the objections urged against the etiological relations of the two affections (which are not necessary to give here, *Tr.*), and then proceeds to establish the relationship on the following grounds:

I. The absolutely established frequency of syphilitic antecedents in cases of locomotor ataxia.

II. The almost exclusive development of tabes in the tertiary period

* Annales de Dermatologie et de la Syphiligraphie, Nos. 1 and 2, 1882.

of syphilis only, as of 85 cases of tabes, 81 began more than four years after the syphilitic infection.

III. The frequent combination with specific syphilitic diseases of the nervous system, of which the following are given :

1. Paralyses of cerebral nerves (optic, nerves of the ocular muscles). According to Ricord, 75 per cent of all paralyses of the eye muscles are syphilitic.

2. Hemiplegic, apoplectiform, epileptiform, aphasic and pseudo-paralytic lesions. Cerebro-spinal tabes results from the combination of the most prominent manifestations of cerebral syphilis with the spinal symptoms.

3. Symptoms of progressive paralysis, the syphilitic origin of which Fournier considers as established.

IV. The (asserted) value of specific medication, especially of iodine, and the uselessness of other remedies in tabes. (Althaus and many others have failed to observe any successful results after specific medication.)

V. The concurrent and intercurrent presence of other undoubtedly syphilitic affections, e.g., exostoses of the femur, tibia and cranium, serpiginous syphilide of the scalp, etc.

VI. The impossibility of showing any other cause for the disease, except syphilis, in 66 cases.

The practical conculsions of Fournier are as follows :

1. In every case of locomotor ataxia, syphilis is to be looked for.

2. Specific treatment is to be instituted in all cases whether syphilis is undoubtedly present or not.

3. As there is danger in delay, treatment should be begun at the very commencement of the tabetic disease. Fournier is confident that he often cures tabes in syphilitics without his knowledge, i.e., by removing the early symptoms, ptosis, amblyopia, etc.

4. Every case of syphilis must be treated energetically and for a long time, because locomotor ataxia is almost always a consequence of neglected or insufficiently treated syphilis.

This review of the recent literature of the relations of locomotor ataxia to syphilis leaves the question still unsettled. The practical importance of the conclusions of Fournier must be, of course, evident. Whether in the initial stages of ataxia in cases with antecedent syphilis, an energetic specific course of medication will prevent the development of the disease, must be answered by further experience. The physician will decide according to his own views whether he will adopt specific treatment in the absence of all syphilitic symptoms. In advanced cases of tabes experience has shown the deleterious effects of antisyphilitic treatment.

